# Apparent absence of avian malaria and malaria-like parasites in northern blue-footed boobies breeding on Isla Isabel

**DOI:** 10.1038/s41598-022-11075-1

**Published:** 2022-04-27

**Authors:** Federico Roldán-Zurabián, María José Ruiz-López, Josué Martínez de la Puente, Jordi Figuerola, Hugh Drummond, Sergio Ancona

**Affiliations:** 1 Departamento de Ecología Evolutiva, Instituto de Ecología, Universidad Nacional Autónoma de México, Mexico City, 04510 Mexico; 2 Facultad de Estudios Superiores Zaragoza, Mexico City, 09230 Mexico; 3 Estación Biológica de Doñana (EBD-CSIC), 41092 Sevilla, Spain; 4 Universidad de Granada, 18071 Granada, Spain; 5 Ciber de Epidemiología y Salud Pública (CIBERESP), 28029 Madrid, Spain

**Keywords:** Ecology, Ecological epidemiology

## Abstract

Haemosporidian parasites are common in birds but are seldom reported in seabirds. The absence of vectors or genetic resistance to infection have been proposed to explain this pattern. However, screening of blood parasites in many seabirds has been done only by visual inspection of blood smears, which can miss low-intensity infections, and molecular detection of blood parasites must be supported by detection in blood smears to confirm the presence of haemosporidians and avoid false positive cases. Here, we tested for the presence of blood parasites of the genera *Plasmodium*, *Haemoproteus* and *Leucocytozoon*, combining inspection of blood smears and PCR-based detection methods in a highly philopatric colony of blue-footed boobies (*Sula nebouxii*) in the Tropical North Pacific. Our results indicate that adults in this colony are likely free of these blood parasites, probably due to unsuitable conditions for insect vectors in booby breeding sites, although potential genetic resistance of blue-footed boobies to infection deserves examination. Apparent absence of blood parasites in Isla Isabel boobies indirectly adds to the growing evidence of variation in parasite infections among avian host species that coexist locally.

## Introduction

*Plasmodium*, *Haemoproteus*, and *Leucocytozoon* are widespread parasites that cause avian malaria and avian malaria–like diseases^1^ and have deleterious impacts on wild birds^2–4^. Infections by these parasites are not homogeneously distributed among bird taxa, and some groups, including seabirds, usually show an extremely low prevalence or total absence of parasite infections^5^. Different hypotheses have been proposed to explain these patterns including that in seabird habitats conditions are unfavorable for vectors^6^. Seabird habitats usually have high salinity, high wind exposure and low vegetation cover, which could reduce the presence of vectors^6,7^. However, even where seabirds and potential vectors co-occur, blood parasite infections are still uncommon^5^, suggesting that alternative hypotheses may explain the observed patterns. Scarcity of infection in seabirds could also be due to good immunological resistance to infections, brief exposure to infections, or lack of suitable host-parasite assemblages^5,8,9^.

**Figure 1:**
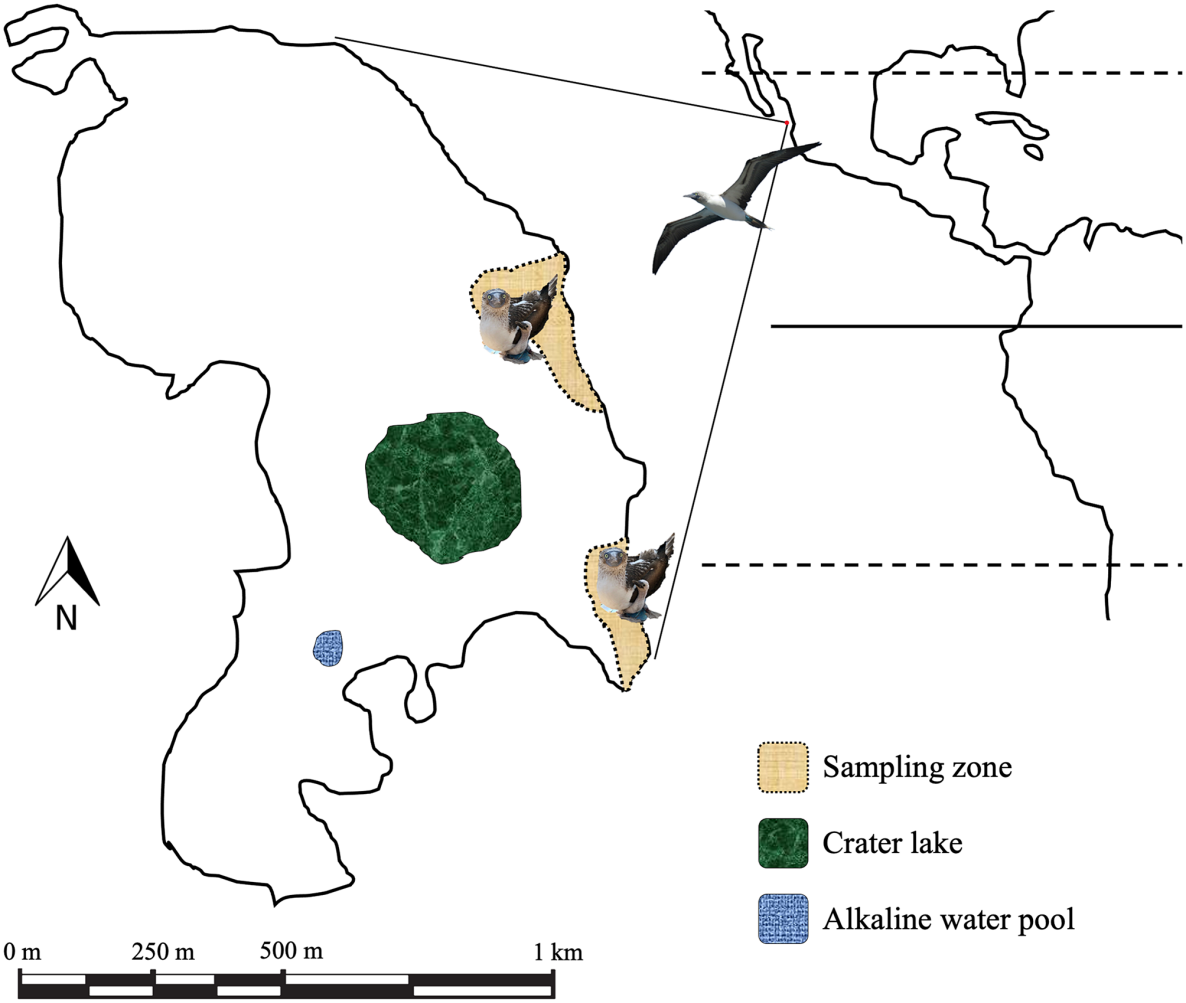
Isla Isabel, Mexico (21° 52′ N, 105° 54′ W) in the Eastern Tropical North Pacific. The study area comprised two fixed plots that measured in total 26,889 m^2^, where reproduction of blue-footed boobies is monitored every year since 1981. Blood samples were taken in March–April 2019. Photos courtesy of FRZ.

Blue-footed boobies are socially monogamous seabirds^10^ that breed colonially on islands of the Eastern Tropical Pacific Ocean, from Mexico’s Gulf of California to northern Peru^11^. Birds nest on the ground in open terrain or in areas with moderate vegetation cover^11^. Isla Isabel (Mexico; Fig. 1) boobies show lifetime fidelity to their first breeding site in their natal colony^10,12^ and may live 20 years or longer^13^. Previous screening of blood parasites in smears of blue-footed boobies from the Galapagos islands revealed the presence of a parasite tentatively identified as *Leucocytozoon* spp.^14^.

Here, we combined microscopic inspection of blood smears and molecular screening to assess the prevalence, and identify genetically, blood parasites belonging to the genera *Plasmodium*, *Haemoproteus* and *Leucocytozoon* in adult blue-footed boobies. We expected Isla Isabel boobies to be infected by blood parasites for two reasons. First, several species of mosquitoes belonging to the genera *Aedes*, *Anopheles*, *Culex* and the potential vectors of *Haemoproteus* and *Leucocytozoon*, including *Culicoides*, hippoboscids and black flies^15^, are widely distributed in Mexico and have been recorded in the study area (^16,17^, authors per. obs.). Second, the prevalence of *Haemoproteus iwa* in Isla Isabel frigatebirds, likely vectored by hippoboscid flies^18^, has been estimated to be 16% (n = 251 frigatebirds^19^), reinforcing the idea that there are suitable insect vectors of haemoparasites on the island.

## Results

No blood parasites were visually detected in 64 blood smears from 33 male and 31 female blue-footed boobies of ages 7 to 13 years that were caring for a clutch or brood, and none of the 64 samples tested molecularly showed evidence of parasite DNA amplification.

## Discussion

Failure to find haemosporidian parasites of the genera *Plasmodium*, *Haemoproteus* and *Leucocytozoon*, using microscopic screening of blood smears and a broadly used PCR-based detection method suggests that breeding male and female blue-footed boobies on Isla Isabel are likely free of these blood parasites. Our results are consistent with Clark and Swinehart’s (1969)^20^ failure to find these parasites in blood smears from 19 blue-footed boobies of Mexico (the number of samples per site is not specified, but the samples were taken on different islands, including Isla Isabel, Todos Santos, Cedros, Cabo San Lucas, San José del Cabo, Cerralvo, Isla Partida, Espíritu Santo, Islas Marietas, Socorro, San Benito and San Martin). These findings are further evidence of the scarcity of blood parasites in seabirds, which is often attributed to unsuitable conditions for vectors in seabird habitats^5,21^.

*Haemoproteus* parasites were detected in Isla Isabel frigatebirds in 1999–2001 (prevalence ranging from 16 to 54%^19,22^). Assuming they were still present in 2019, lack of blood parasites in our sample implies that presence of parasite infections varies among Isla Isabel’s seabird species.

The difference between boobies and frigatebirds in the prevalence of blood parasites could be due to spatial variation in the abundance and activity of vectors within the island^23,24^. Infected frigatebirds were sampled in the southwestern end of the island, in a shady, vegetated area with large trees 40 m from a concrete building where rainwater accumulates, and 150 m from the only pool of alkaline water on the island^19,25^. This area is protected from the wind and suitable for development and proliferation of some potential vectors^26^. Boobies breed mostly ~ 1.0–1.5 km away, at the wind-exposed northeastern end of the island, where we sampled them. The latter area is mainly covered by stunted garlic pear trees recurrently damaged by hurricanes^27,28^, and the hot and dry conditions there may limit the abundance and activity of potential vectors of blood parasites^6,23^, explaining the absence of blood parasites in our sample. In addition, the difference between boobies and frigatebirds in the prevalence of *Haemoproteus* parasites could be due to differences in the sampling years if parasites found two decades ago are no longer found in either species now.

Alternatively, the absence of blood parasites in blue-footed boobies despite the presence of *Haemoproteus* parasites in frigatebirds, could be due to strong specificity between the parasite or the vector and their vertebrate host^29,30^. The blood parasites detected in Isla Isabel frigatebirds belong to the morphospecies *Haemoproteus iwa*^19^, which is highly prevalent on frigatebirds and potentially specializes in parasitizing them^18,22,30^. *H*. *iwa* is transmitted by louse flies (Hippoboscidae)^30^. These blood-sucking insects dwell mainly on their vertebrate hosts and present higher host specificity than mosquitoes, *Culicoides* and black flies. However, specificity of louse flies may be low and they could potentially transmit *Haemoproteus* parasites to Isla Isabel boobies, since louse flies are often reported on boobies^31,32^ and have been anecdotally observed during the manipulation of blue-footed boobies on Isla Isabel (authors per. obs.^33^). Then, strong host specificity of the parasite not the vector could preclude transmission of *Haemoproteus* parasites to Isla Isabel boobies.

Haemosporidians have earlier been detected in southern populations of blue-footed boobies^14,18^ but these observations are under debate^34^ and these boobies could be free of blood parasites along their geographic range. Galapagos boobies were found to be infected by *Haemoproteus* parasites using PCR^14,18^. Levin et al.’s identification (2011)^18^ of *Haemoproteus* spp. may have been a result of PCR contamination or confusion among samples rather than a true infection. The lineage of *Haemoproteus* spp. found by Levin et al. (2011) matched a sequence obtained in the same laboratory from a cirl bunting *Emberiza cirlus* in southeast Europe, raising concerns of cross-contaminations during the analyses^34^. Further confirmatory PCR essays of boobies’ samples tested negative for avian haemosporidians and potential cross-contaminations could not be ruled out since blood smears were not collected in parallel with blood for molecular screening, which is required to confirm the presence of haemosporidians and discard such false positive cases^9,34^. Lee-Cruz et al.’ s detection (2016)^14^ of *Haemoproteus* spp. by PCR of individuals with negative blood smears was attributed to low levels of parasitaemia or detection of free DNA rather than viable parasites, but the discrepancy could be an indicator of potential PCR contamination^34^. Only a parasite tentatively identified as *Leucocytozoon* spp. was detected using blood smear screening^14^. Thus, further analyses of blue-footed boobies of the Galapagos and further south (e.g., Peru) are needed to confirm infection by avian haemosporidians.

The absence of blood parasites in boobies despite previous detection of potential vectors in the area could also be explained by overly short exposure to parasitic infections, physiological incompatibility with haemoparasites that prevents them from completing their life cycles, or high immunological capacities^8,9^. Moreover, genetic differences between blue-footed booby populations^35^ could cause differences in immune capacity and prevalence of blood parasites. Studies comparing immunocompetence among populations of the same species are still scarce, but data for fish^36^, birds and mammals^37,38^ suggest that this possibility deserves examination.

Importantly, high and rapid mortality after blood parasite infection may occur in naïve avian host populations^39,40^, drastically reducing the prevalence of parasites in sampled birds. However, it is unlikely that this explains the apparent absence of blood parasites in the Isla Isabel boobies, since haemosporidians were detected on the island twenty years earlier^19^. Similarly, it is unlikely that a sample of 64 birds in a single year yielded a biased estimate of blood parasite prevalence in Isla Isabel boobies. Firstly, sample sizes above 15 individuals are expected to produce robust prevalence estimates^41^. Secondly, although environmentally driven inter-annual variation in the prevalence of blood parasites is common^42,43^, antagonistic interactions between bird hosts and blood parasites tend to be stable over time^44^. Nevertheless, additional screening of birds in other years is desirable to fully confirm the absence of blood parasites in this booby colony.

In conclusion, we failed to find evidence of blood parasites in one of the largest colonies of blue-footed boobies of the North Pacific coast, after microscopic examination of blood smears and state-of-the-art molecular analysis for detection of avian blood parasites^9,45^. Apparent absence of blood parasites in Isla Isabel boobies indirectly adds to the growing evidence of variation in parasite infections among avian host species that coexist locally^23,46^, and highlights the relevance of performing evaluations of the prevalence of blood parasites in different populations of widespread host species^21^.

## Methods

### Study site

Isla Isabel is an 82-ha volcanic island 28 km off the west coast of Mexico, in the Eastern Tropical North Pacific (21°52′ N, 105°54′ W). The island is mainly covered by deciduous dry forest of *Crataeva tapia* trees, *Euphorbia schlechtendalli* bushes, and coastal grasslands. The climate is sub-humid tropical with rains in June–November (the hurricane season). In the rainy season, water is collected in three endorheic basins in the center and north of the island. There is also a shallow pool of alkaline water of approximately 50 m in diameter in the south of the island that is the result of rainwater runoff and the entry of seawater during storms^27^. The booby colony of Isla Isabel has up to 1769 breeding pairs in our study area alone, which covers 26,889 m^2^ and contains approximately 65% of all breeding pairs on the island^47^.

### Field procedures

In March–April 2019, we hand-captured 64 adult blue-footed boobies (33 males and 31 females) on their nests (sites with a clutch or brood) at night. We recorded the identity of boobies that had a metal ring (since 1989, tens of thousands of fledglings and adults have been banded on Isla Isabel^13^) and sexed all captured boobies by voice (females grunt, males whistle). Our sample included 28 breeding boobies banded as fledglings aged from 7 to 12 years in females and 8 to 13 years in males. We captured adults that had been incubating a clutch (17 males and 16 females) or caring for a brood (16 males and 15 females) for at least 15 days, to minimize the risk of adults abandoning their clutches and milksnakes preying on chicks. See Drummond et al.^48^ for further details on the field procedures used.

Approximately 1.5–2.0 ml of blood were taken from each adult’s brachial vein. A drop of blood was used for blood smear preparation and 200 µl were split in two aliquots (100 µl each) and stored in 96% ethanol for molecular screening of blood parasites. The remaining blood was centrifuged and stored for future research. Blood smears were fixed in 96% ethanol and subsequently stained with Giemsa. Manipulation of booby adults took less than 10 min and bleeding stopped before release at the site of capture. All adults resumed nest attendance 5–10 min after release. Data collection and blood sampling protocols comply with the current laws and ethical standards of animal research in Mexico (NOM-059-SEMARNAT-2010) and were revised and approved by the Secretaría de Medio Ambiente y Recursos Naturales (SEMARNAT; permit number SGPA/DGVS/01216617). We confirm that all methods are reported in accordance with ARRIVE guidelines 2.0 (https://arriveguidelines.org).

### Parasite identification

Blood smears were scanned for the presence of blood parasites using a light microscope Nikon Eclipse Ti—Arcturus XT of Applied Biosystems. Half of each blood smear was scanned at 400× magnification in search of larger parasites, including *Leucocytozoon*, during 30–45 min. The other half of each smear was scanned at 1000× magnification in search of *Haemoproteus*, *Plasmodium* and *Leucocytozoon* parasites in up to > 10,000 erythrocytes per smear (see^49^) during 30 min.

DNA from blood samples was extracted using the Maxwell^®^ 16 LEV system Research (Promega, Madison, WI)^50^. Samples were run in 0.8% agarose gels to check DNA integrity. To detect and identify avian parasites of the genera *Plasmodium*, *Haemoproteus* and *Leucocytozoon*, we carried out two nested PCRs following Hellgren et al.^51^ to amplify a 478 bp fragment of the mitochondrial cytochrome b gene. For the first PCR we used primers HaemNFI (5′-CATATATTAAGAGAAITATGGAG-3′; I = a universal base, inosine) and HaemNR3 (5′-ATAGAAAGATAAGAAATACCATTC-3′) to amplify parasite mtDNA from the three genera of avian malaria parasites. For the second PCR, we used HaemF–HaemR2 primers^52^ for *Plasmodium* spp. and *Haemoproteus* spp., and primers HaemFL (5′-ATGGTGTTTTAGATACTTACATT-3′) and HaemR2L (5′-CATTATCTGGATGAGATAATGGIGC-3′) for *Leucocytozoon* spp.^51^. The first PCR (including HaemNFI–HaemNR3) was performed in 25 µl reactions with 2 µl of DNA (containing around 50 ng of total genomic DNA), 1× PCR Buffer, 2.5 mM MgCl2 and 0.6 Units of Taq Polymerase (BIOTAQTM DNA polymerase, Bioline), 0.3 mM dNTPs (Bioline), 0.33 mg/ml of BSA (Roche Diagnostics) and RNase-free water. The PCRs were conducted using the following conditions: 45 s at 94 °C, 45 s at 50 °C, and 1 min at 72 °C for 34 cycles. The samples were incubated before the cyclic reaction at 94 °C for 3 min and after the cyclic reaction at 72 °C for 10 min. We used 2 μl of the first PCR reaction as the template for the second PCR, 2 μl for *Leucocytozoon* spp. (HaemFL–HaemR3L) and 2 μl for *Haemoproteus* spp.–*Plasmodium* spp. (HaemF–HaemR2). These PCRs were performed separately in 25-μl volumes with the same proportions of reagents as in the initial PCR reactions and using 0.15 mg/ml of BSA (Roche Diagnostics). The thermal profile of the PCR was identical to the initial PCR. Each PCR contained a positive sample and a negative control. To check if the PCRs amplified successfully, we ran 3.0 μl of the final PCR product on a 2% agarose gel. We ran six repetitions of each PCR to exclude false negatives.

### Ethics approval

Our research complies with Mexican legal and ethical requirements. Permit to collect blood samples (SGPA/DGVS/01216617) was provided by Secretaría del Medio Ambiente y Recursos Naturales (SEMARNAT).

## Supplementary Information

Dataset S1.

Supplementary Information.

## Data Availability

All data generated during this study are included in this published article [and its supplementary information files].
